# Understanding AMD by analogy: systematic review of lipid-related common pathogenic mechanisms in AMD, AD, AS and GN

**DOI:** 10.1186/s12944-017-0647-7

**Published:** 2018-01-04

**Authors:** Qinyuan Xu, Sijia Cao, Sanjeeva Rajapakse, Joanne A. Matsubara

**Affiliations:** Department of Ophthalmology and Visual Sciences, Faculty of Medicine, University of British Columbia, Vancouver, BC V5Z 3N9 Canada

**Keywords:** Lipids/oxidation, Cholesterol, Apolipoproteins, Inflammation, Complement, Macrophages, Diseases

## Abstract

**Rationale:**

Age-related macular degeneration (AMD) is one of the leading causes of blindness among the elderly. Due to its complex etiology, current treatments have been insufficient. Previous studies reveal three systems closely involved in AMD pathogenesis: lipid metabolism, oxidation and inflammation. These systems are also involved in Alzheimer’s disease, atherosclerosis and glomerulonephritis. Understanding commonalities of these four diseases may provide insight into AMD etiology.

**Objectives:**

To understand AMD pathogenesis by analogy and suggest ideas for future research, this study summarizes main commonalities in disease pathogenesis of AMD, Alzheimer’s disease, atherosclerosis and glomerulonephritis.

**Methods:**

Articles were identified through PubMed, Ovid Medline and Google Scholar. We summarized the common findings and synthesized critical differences.

**Results:**

Oxidation, lipid deposition, complement activation, and macrophage recruitment are involved in all four diseases shown by genetic, molecular, animal and human studies. Shared genetic variations further strengthen their connection. Potential areas for future research are suggested throughout the review.

**Conclusions:**

The four diseases share many steps of an overall framework of pathogenesis. Various oxidative sources cause oxidative stress. Oxidized lipids and related molecules accumulate and lead to complement activation, macrophage recruitment and pathology. Investigations that arise under this structure may aid us to better understand AMD pathology.

## Background

Age-related macular degeneration (AMD) has become the third leading cause of blindness within developed countries and among the elderly, ranked after cataract and glaucoma [1]. Due to its complex etiology, current therapeutic approaches have been insufficient at treating early AMD. Previous studies of AMD reveal three systems that are closely associated with its pathogenesis: lipid metabolism, oxidative stress and the inflammatory process. Interestingly, these same three systems are also involved in Alzheimer’s disease (AD), atherosclerosis (AS) and glomerulonephritis (GN). In addition, general risk factors such as advanced age, smoking and specific genetic variations are shared between AMD and these three diseases [2–6]. Most importantly, each disease has lipid-rich deposits that are characteristic of their pathology. Together, these commonalities lead us to investigate the shared pathogenic mechanisms among the four diseases, which may provide insight into those aspects of AMD development that are still unclear. The scope of this paper includes deposition of lipids and lipoproteins and their consequences in the four diseases.

### Age-related macular degeneration

Age-related macular degeneration (AMD) has quickly become the leading cause of vision impairment for the elderly in developed countries. While early and intermediate AMD do not usually cause symptoms, late AMD can cause severe central vision loss. Late AMD affects the macular region on the retina and is divided broadly into two types. Nonexudative (dry) AMD is characterized by drusen, a type of lipid-rich extracellular deposit. Advanced nonexudative AMD, called geographic atrophy, involves a slow deterioration of the retinal pigment epithelium (RPE) and secondary photoreceptor loss. Exudative (wet) AMD is characterized by choroidal neovascularization. Although this form of AMD is less common than nonexudative AMD, its onset is more acute and causes 90% of all cases of severe vision loss due to AMD. Currently, there are no treatments for early AMD and its etiology has yet to be fully characterized. A multitude of risk factors have been recognized. Aging, smoking and genetic predisposition are found to be common amongst AMD patients [3, 7, 8]. Pathological processes such as lipid metabolism, oxidative stress and inflammation are suggested to be closely involved in AMD pathogenesis. Recently, Pujol-Lereis and colleagues suggested a reasonable pathogenic mechanism that links the three systems [9]. Under oxidative stress, lipid deposits called drusen form in the retina and trigger chronic inflammation by activating the complement system. Then, immune cells such as macrophages facilitate more severe pathogenesis. These events eventually cause RPE cell death and central vision loss.

### Alzheimer’s disease

Alzheimer’s disease (AD) is a progressive neurodegenerative disease and the most common form of dementia in older adults. Clinically, AD patients first develop an inability to form recent memories, which then progresses to a stage of dementia that affects all cognitive functions. Later in the disease course, AD patients commonly rely on caregivers for basic activities of daily living, and many have shortened life spans. Clinical examination, lumbar puncture or PET imaging studies can diagnose AD, although each has its own drawbacks such as specificity or accessibility. Brain structures such as the hippocampus and cerebral cortex are involved, resulting in progressive inability to consolidate memories and perform higher functions such as decision making. Pathologically, hallmarks of AD include intracellular neurofibrillary tangles and extracellular amyloid-β (Aβ) protein plaques, accompanied by reactive microgliosis, dystrophic dendrites and axons, loss of synapses and neuronal degeneration [10]. It has been widely reported that lipid homeostasis around cholesterol, oxysterol and apolipoproteins are also crucially involved in AD pathogenesis [11, 12].

### Atherosclerosis

Atherosclerosis (AS) is a degenerative process that involves inflammatory lesions of the arterial walls. It can affect larger blood vessels such as the carotid artery or smaller vessels such as the coronary artery. Depending on the type of blood vessels involved, it increases the risk of stroke and cardiovascular diseases such as myocardial infarction. While the incidence of stroke has declined globally, the incidence of AS-related heart disease increased particularly in Eastern Europe and Asia [13]. Apart from ischemic brain and heart disease, AS can also affect the peripheral vascular system resulting in claudication, or gastrointestinal vascular system. Some may have severe consequences such as ruptured abdominal aortic aneurysms. Diet, smoking, exercise and genetic risk factors have been identified for AS and other cardiovascular diseases. In AS, lipoproteins and lipids cross endothelium of large arteries and deposit in the arterial intima. Eventually these deposits form atherosclerotic plaques inside the arteries and cause further damage to the cardiovascular system by inducing chronic inflammation [14].

### Glomerulonephritis

Glomerulonephritis (GN) is a term used to describe the group of diseases affecting glomeruli in the kidneys. There are many subtypes of GN. This paper will focus primarily on Type II membranoproliferative glomerulonephritis (MPGN II), with a brief mention of relevant studies in other GN subtypes. Although MPGN is an uncommon cause of chronic nephritic disease, literature supports that MPGN is a global phenomenon. It has been reported to be a common form of primary glomerular disease in Peru and Africa [15, 16]. In the US and Japan, many cases of MPGN are associated with chronic hepatitis C virus infection [17, 18]. Interestingly, epidemiology of MPGN is variable throughout different parts of the world, likely due to differences in genetics as well as environmental exposure. Clinically, MPGN results in severe proteinuria and end-stage renal disease. MPGN may be idiopathic or a secondary manifestation of a systemic disease. The idiopathic MPGNs are further divided into three subtypes. Type I is characterized by subendothelial deposits, type II is characterized by dense deposits in the glomerular basement membrane and type III is characterized by subepithelial and subendothelial deposits. Major pathological events in MPGN II include the presence of a hypercellular kidney mesangium and electron dense material accumulating in the glomerular basement membrane. Lipid dysregulation in the kidneys has been used as analogy to study and understand other diseases such as AS and systemic AA amyloidosis [19, 20]. The lipid nephrotoxicity hypothesis suggests that kidney diseases involve many of the similar processes in AMD. These include problems in lipid transport, which then leads to lipid relocation and accumulation in different diseased tissues [19].

## Oxidation and lipid-rich deposits

### Oxidative stress

AMD, AD, AS and GN are lipid-related diseases in which extracellular lipid-rich deposits, lipid-related proteins, and oxidized lipids play a pathological role. The source of oxidation varies between the four diseases. Photo-oxidation is uniquely applicable to eye diseases. Previous studies correlate high exposure to sunlight or UV light to the development of AMD [21, 22]. Reactive oxygen species (ROS) related oxidative processes happen throughout the body, but more prone to the eyes, the brain and the kidneys. These organs are known to have high and constant blood supply, which exposes them to high oxygen partial pressures that increase the likelihood of ROS production. In AMD, with the highly oxidative environment in the retina, ROS is produced in the RPE cells [23]. It is also widely understood that ROS can cause lipid peroxidation and serves as an important inducer of AS development [24]. In vitro experiments using human endothelial cells confirmed the existence of proatherosclerotic oxidative stress from ROS produced by NADPH oxidase [25]. Since chemical exposure from cigarette smoking is an important risk factor for both AMD and AS, and currently it is unknown whether the oxidants in cigarettes reaches the eye, further investigation should be done to see if cigarette smoking result in excessive ROS production in the eyes. Certain toxin-related oxidation is uniquely involved in AD. It has been reported that pathologic protein aggregates such as Aβ peptide, α-synuclein, and tau-441 protein induce oxidative stress and over production of ROS [26, 27]. Following this framework of toxin induced ROS production in AD pathology, combined with the observation that very low density-like lipoproteins (VLDL) are produced and secreted by RPE [28], it is reasonable to next investigate whether VLDLs induce ROS formation in RPE. To our best knowledge, oxidative stress in MPGN II is not well studied, but oxidative stress plays an important role in kidney damage. In human renal glomerular endothelial cells, advanced oxidation protein products induce endoplasmic reticulum stress and endothelial-to-mesenchymal transition [29]. In a transgenic mouse model of focal segmental glomerular sclerosis, renal podocytes secreted endothelin-1, which induced mitochondrial oxidative stress in renal endothelial cells [30]. Interestingly, plant extract from Cordyceps militaris reduced oxidative stress and inflammation by normalizing nuclear factor kappa B activity. This resulted in recovery of kidney function and histological architecture in a rat model of membranous glomerulonephritis, a subtype of GN related to MPGN [31]. These studies suggest an important role of oxidative stress in MPGN and related pathologies.

### Lipid-rich deposits

In addition to oxidative stress, accumulation of lipid-related molecules is another common factor seen in AMD, AD, AS and GN. Along with other molecules, oxidized lipids and lipid-related molecules accumulate in the Bruch’s membrane (BrM) of retina, brain parenchyma, arterial intima, and glomerular basement membrane (GBM).

The deposits found in the retina are called drusen, an extracellular deposit rich in lipid and protein [32]. In 2010, Wang et al. reported that more than 40% of drusen volume is composed of lipid-containing particles [32]. There are several types of drusen with different morphology and contents [33]. Soft drusen have homogenous content and exist in the macula region, while hard drusen have substructures and appear in both macular and peripheral regions [33, 34]. Other types of clinically detectable drusen include cuticular drusen, calcified drusen, reticular pseudodrusen and “ghost drusen” [34]. For a comprehensive review of the different types of drusen, please refer to [34]. The exact mechanism of drusen formation remains unclear to this day, but theories exist for the process of cell damage due to lipid deposition. Preceding drusen formation, BrM thickens due to accumulation of lipids, heterogeneous material and inflammatory debris, which slows down nutrient and waste transportation between RPE cells and capillary lamina of choroid, leading to RPE cell damage [35]. Recently, Thompson et al. proposed a novel mechanism of drusen biogenesis based on hydroxyapatite spherules [36]. First, natural lipid droplets form at the RPE/choroid interface. Then, insoluble hydroxyapatite shells form around them. Finally, proteins and lipids attach to hydroxyapatites and initiate drusen formation [36].

Lipid-rich deposits like drusen are found in the other three diseases as well. In AD, the deposits found in the brain parenchyma are called senile plaques (SP). SPs are round masses in the brain composed of amyloid peptides, oxidized proteins and lipids. In AS, atherosclerotic plaques are formed when lipids cross the blood endothelium into the intima of the arteries. Since the 1990s, it has been established that atherosclerotic plaques contain oxidized lipids, as well as other components such as tocopherol and ascorbate [37]. The deposits formed in MPGN are electron dense deposits that sit in the GBM and are diagnostic of MPGN II [38]. While the exact composition of the electron dense deposits remains unclear, they are distinctively different from normal GBM under histological examination [39]. Interestingly, patients with MPGN II also develop ocular lesions that are indistinguishable from AMD [40]. Given this important commonality of lipid-rich depositions between the four diseases, future studies should pay attention to the compositions of these deposits in order to find worthwhile therapeutic targets common to multiple diseases.

### Lipid-related molecules

Although there are many lipid-related molecules involved in the pathogenesis of AMD, AD, AS and GN, not all of them are involved in oxidation. Some of the most common lipid-related molecules involved in oxidation and lipid deposition include oxidized cholesterols, apolipoproteins (apo), and oxLDL.

#### Oxidized cholesterols

Oxidized cholesterols are a category of molecules that has been extensively studied. While oxidized cholesterols are involved in AMD, AD and AS, they do not appear to be significantly involved in GN. 7-ketocholesterol (7kCh) is one of the important oxidized cholesterols involved in AMD. 7kCh deposits has been found at BrM, choriocapllaris and endothelial cell surfaces [41]. It induced vascular endothelial growth factor production in RPE cell cultures and has been linked to choroidal neovascularization in the wet form of AMD [41]. In AD, 7kCh sensitizes the cell membranes to interact with Aβ, an important peptide in AD pathogenesis, although the interaction was not strong enough to induce downstream pathogenic events such as intracellular transfer of the peptide [42, 43]. K^+^ homeostasis disruption, another contributor to AD pathogenesis, is also triggered by 7kCh [44]. Given these functions of 7kCh in AD, it will be interesting to see if 7kCh sensitizes RPE cell membrane’s interaction with other lipid-related molecules, such as apo, or if it disrupts ion channels in RPE cell membranes. There are hundreds of papers in the literature suggesting 7kCh’s association with AS. Many explore the cellular mechanisms underlying the damage induced by 7kCh. It was repeatedly shown that 7kCh efficiently induced apoptosis and cytotoxicity in endothelial cells [45, 46]. Related to cell damage, 7kCh was capable of inducing intracellular ROS production in endothelial cells [46]. Since ROS production in RPE cells is associated with AMD, it is worthwhile to investigate if oxidized cholesterol molecules induce ROS production in RPE cells. Further, it has been reported that in vascular smooth muscle cells, 7kCh treatment is able to increase cholesterol levels in a dose-dependent manner [47]. Given that cholesterol is the major component of AS plaques, 7kCh may be an important inducer of AS plaque formation.

Cholesterol may also be oxidized on the side-chains. One of these molecules, 24-(S)-hydroxycholesterol (24S–OHC), is involved in both AMD and AD. In AMD, they are capable of causing increased Aβ production, ROS production, inflammation and apoptosis in a RPE cell line [48]. In AD patients, higher plasma levels of 24S–OHC were found compared to controls [49]. In murine oligodendrocytes, which work closely with brain neurons, both 7kCh and 24S–OHC can trigger K^+^ homeostasis disruption, suggesting a pathogenic role of these two molecules in AD [44]. Both 7kCh and a close relative of 24S–OHC, 25-hydroxycholesterol (25OH), were found to enhance morphological cellular changes after the cell membrane associates with Aβ [42]. In another study, while 7kCh could not elicit more cellular response than increased cell membrane-Aβ association, 25OH was capable of inducing membrane raft-dependent transport of Aβ in Jurkat cells [43]. This suggests that hydroxycholesterols might be a more potent inducer of cellular changes. Interestingly, this type of oxidized cholesterol was also reported to be a remedy to pathology. In human neuroblastoma cells, 24S–OHC treatments protected the cells from 7kCh-induced cytotoxic cell death [50]. This interesting evidence in AD suggest that investigations into the role of these side-chain oxygenated cholesterol may help us to understand RPE cellular changes in AMD.

#### Cholesterol

Cholesterol itself is associated with the lipid deposits, although the role of non-oxidized cholesterol in disease development is unclear. Only a small number of studies reported a significant increase in AMD risk with high levels of total cholesterol [51]. Some studies suggest no significant association between total cholesterol and AMD, while others show an inverse relationship [51]. A mouse model showed that lipoprotein accumulation and cholesterol esters presence in the BrM caused the development of structural changes that signifies aging, but not to the extent where AMD develops [52]. A rat model demonstrated that while 7kCh ocular implants caused massive angiogenesis and inflammation, cholesterol implants caused no angiogenesis and very little inflammation [53]. In AD, while 7kCh enhanced the cell membrane’s interaction with Aβ in cell-sized liposomes, cholesterol inhibited this crucial event in AD pathogenesis [42]. Therefore, non-oxidized cholesterol may not have an impact on the pathogenesis of these diseases.

#### Apolipoproteins

As previously described, lipids and proteins are the major components of drusen [32]. Related to lipid molecules, apolipoproteins (Apo) are also important components of the lipid-rich deposits. Lipid metabolism is governed by Apo, which are structural proteins that are integral to the metabolism of triglycerides and cholesterol in the body. In AMD, RPE secretes Apo into the BrM, which accumulates with aging and forms a lipid barrier that retards the transportation of oxygen, nutrient and waste products [51]. Importantly, this lipid barrier is the cradle for drusen formation. In AD studies, immunostaining experiments revealed that apolipoprotein E (ApoE) and cholesterol co-localizes with Aβ in SPs of mouse models [54]. Other immunoreactivity experiments showed the presence of ApoE, low-density lipoprotein (LDL) receptor and LDL receptor related proteins in SPs [55]. Further, Sun and colleagues directly suggested that preferential uptake of oxidized lipoproteins might function as an oxidative stressor itself and initiate neuronal cell death to cause AD [56]. Apo is also found in atherosclerotic plaques. The “response to retention” hypothesis of atherosclerosis suggest that subendothelial lipoprotein retention initiates AS pathology [57]. The same hypothesis has been used to describe similar steps in AMD pathogenesis [58]. In AS and AMD, shared lipoprotein by-products, such as linoleate hydroperoxide and 7kCh, are identified in the aortic intima and the BrM [41, 59]. In MPGN II, Apo have been found in the microdissection samples of MPGN II with 100% probability by mass spectroscopy [38].

Furthermore, Apo is an important constituent of LDL, composed of mainly cholesterol and protein along with other lipid-related molecules. Specifically, oxidized LDL (oxLDL) has been found in lipid deposits of both AMD and MPGN. Immunohistochemistry studies revealed the presence of oxLDL in surgically removed choroidal neovascular membranes from eyes of AMD patients [60]. In mesangial matrix of kidney, accumulation of oxLDL can cause cytotoxicity, increase the cross-linking of glycated matrix components and establish conditions that favour mesangial sclerosis [61]. Given the evidence of Apo’s involvement in all four diseases, it is possible that Apo is a lipid-related molecule universally involved in many oxidation and inflammation-related diseases. Investigations into targeted treatment to lower Apo or regulate Apo secretion from certain cells, such as RPE cells, may yield effective treatment that works for multiple diseases.

Genetic variations in genes regulating lipid metabolism, such as the APOE gene coding for apolipoprotein E, constitute important risk factors for AMD, AD, and AS. The APOE-4 allele was associated with a decreased risk of AMD, while the APOE-2 allele was associated with an increased risk of AMD for males in population studies [62, 63]. Differences are apparent among the other diseases studied here, as in AD it is well established that APOE-4 allele is the most prevalent risk factor [64]. Both late-onset familial AD and sporadic AD have increased prevalence of APOE-4 alleles compared to controls [55]. Mechanistically, the protein coded by APOE-4 binds to Aβ and prevents Aβ from being degraded by neprilysin, thus promoting its aggregation [2]. Furthermore, APOE-4 interacts with cerebral spinal fluid (CSF) amyloid and CSF hyperphosphorylated tau protein (ptau) and exerts a synergistic relationship with complement protein C3 to cause pathology and neurodegeneration [65]. Although we understand some of the mechanisms in which Apo work, one question still lingers. Why would different types of Apo have such contrasting effects in related diseases such as AMD and AD?

In AS, gene variants related to atherosclerosis through carotid intimal-medial wall thickness include genes related to matrix deposition (MMP3), inflammation (interleukin 6), and lipid metabolism (hepatic lipase, APOE, CETP, and PON1) [Reviewed by [66]]. This evidence supports the interaction between at risk lipid metabolism and inflammatory responses. Other variations in cholesterol-related genes are also important risk factors for AMD. For example, gene variants of cholesteryl ester transfer protein (CETP), hepatic lipase (LIPC), and ATP-binding cassette transporter A1 (ABCA1), are cholesterol-related genes that are involved in AMD [Reviewed by [23]]. These genes code for protein products involved in cholesterol transport and metabolism. Given the variety, complexity and relatedness of lipid metabolism genes, composing a mind map of their relationship will aid the identification of important genes involved in the pathogenesis of lipid-related diseases.

#### CEP

Carboxyethylpyrroles (CEP) are phospholipid-modified protein adducts that are closely involved in the pathogenesis of AMD [67]. In the eyes, CEP accumulates in the RPE and photoreceptor outer segments [67]. In AMD, CEP-modified proteins are present at higher levels in drusen, retina and blood [67]. CEP-modified proteins are also present in the brain, although instead of AD, they are related to autism [67]. In AS, CEP was found in human AS lesions both within and outside macrophages [68]. In mice fed with Western diet, immunofluorescence experiments showed CEP accumulation in their aortic root [68]. Although we know that CEP is associated with oxidative stress and inflammatory signals in macrophages [69], and that both processes are involved in AD and GN, CEP are not specifically associated with these two diseases. Therefore, investigating the role of CEP in AD and GN pathology may yield fruitful results. There are many other types of modified-proteins involved in AMD, such as aldehyde-modified proteins and 4-hydroxynonenal-modified proteins [70, 71]. Since this review focuses mainly on lipid depositions and downstream consequences, they are out of the scope of this review.

#### Bisretinoids

Bisretinoids, components of lipofusin, are another category of lipid-related molecules that plays an important role in AMD. Oxidized bisretinoids directly activate the complement system, most likely through the alternative pathway [72], as shown by in vitro experiments on human RPE cells [73]. Bisretinoids are degraded when short-wavelength light hits the retina and this type of photodegradation is ongoing in the eye [74]. In the eye, photodegradation of certain bisretinoids, such as A2E and all-trans-retinal dimer, generates dicarbonyls glyoxal (GO) and methylglyoxal (MG) [75]. In AMD, these by-products modify proteins by advanced glycation endproduct (AGE) formation [75]. In AS, proteins are also modified by MG and GO by non-enzymatic glycation and oxidation reactions, in addition to AGE formation as in AMD [75]. Just like CEP, bisretinoids are not reported to have significant roles in AD or GN.

## Inflammation

Inflammation plays an important role in AMD, AD, AS and GN. Under normal circumstances, appropriate activation of the inflammatory process help the body to remove waste materials and apoptotic cells, protecting the individual from disease development. For example, the initial purpose of complement activation in AD may be simply to remove Aβ plaques. Without complement activation, AD mice displayed higher Aβ deposition and more severe neurodegeneration when compared to mice of the same age [76]. In AS, the classic complement factor C1q reduces early AS in mouse models [77]. Likewise, macrophages may have a beneficial role in the treatment of AD. In mouse models, weekly injection of macrophage colony stimulating factor (M-CSF), a factor that allows microglia to differentiate into macrophage-like cells, prevented cognitive loss and resulted in smaller and less dense SPs [78].

**Fig. 1:**
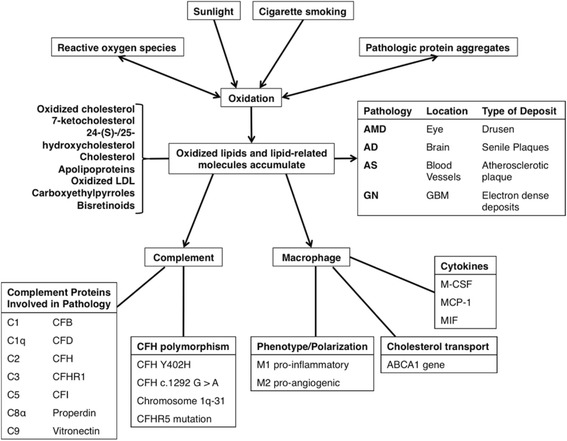
Lipid-related common pathogenic mechanisms in AMD, AD, AS and GN. Various oxidative sources cause lipid-related molecules to undergo oxidation, which then accumulate in the site of disease progression. Depending on the type of deposit, these accumulations lead to different pathologies. Common among the different pathologies, these lipid-rich deposits activate inflammation through complement activation and macrophage recruitment. Many complement proteins and CFH-related polymorphisms are involved in the four diseases discussed in this review. Macrophages are recruited and further differentiated due to the presence of various cytokines as well as lipid transport metabolism

However, excessive and inappropriate inflammation can cause tissue damage and thus inhibiting excessive inflammation may control disease progression. One of these inhibiting factors is complement factor H (CFH). BrM specimens from AMD patients confirmed that the presence of CFH, a major inhibitor of the alternative complement pathway, removes lipoproteins and serves as a protective factor in disease progression [79]. Excessive inflammation in the brain also leads to pathogenesis. When macrophages in the brain fail to clear excess Aβ proteins, they become chronically activated and cause neuronal damage by secreting neurotoxins such as free radicals, nitric oxide, cytokines and N-Methyl-D-aspartic-acid-like molecules [80, 81]. Complement activation and macrophage recruitment are two major inflammatory pathways involved in the pathogenesis of AMD, AD, AS and GN (Fig. 1). We will discuss them in more detail in the following sections.

### Complement activation

In AD, complement factor B and associated by products of the alternative complement system are increased in the frontal cortex of AD patients [82]. Interestingly in AD, C3 and ApoE have a significant interaction on both CSF Aβ and CSF ptau [65]. In contrast to AMD, the inhibitory factor H and factor I were not significantly increased in AD. In a mouse model of AS, mRNA and qPCR analysis showed the presence of alternative pathway components in mice under diet-induced AS conditions [83]. These include C3, properdin, and factor D. Specifically, C3 was found in aortic lesions. In MPGN, components of the alternative complement pathway were identified in almost all dissected glomeruli samples in a 2009 study [38]. Mass spectroscopy experiments identified the presence of complement components, including C3, C5, C8α, C9, Complement Factor H Related Protein 1, vitronectin and ApoE in laser micro-dissections of MPGN glomeruli with 100% probability [38]. The breakdown products of C3, including C3β and C3β chains, are also identified in the dissections and reported to be the components that were buried away in the glomeruli [38]. Together, this evidence supports the close involvement of alternative complement pathway in the four diseases. Although each disease may involve slightly different molecules, regulation of the alternative complement pathway is a common target for future research. The significant molecules involved in related diseases will also serve as a library in which we can target specific molecules to study in AMD models.

Closely functioning together with the alternative complement pathway, genetic variations in CFH also appear to be important in AMD, AS and GN. In human eyes with AMD, CFH co-localizes and binds with oxidized lipids in drusen [84]. Specifically, CFH variant 402Y of the protective rs1061170 genotype have a higher affinity for oxidized lipids and more effective inhibition of oxidized lipid interaction with RPE and macrophages [84]. On the other hand, the CFH risk allele 402H is correlated with a lower capacity to inhibit the induction of the complement system and so increased risk of AMD [84]. This polymorphism seems to be specific to AMD, as scientists are unable to detect an association of Y402H polymorphism with AD, AS, and insufficient literature is established around Y402H and GN [85, 86]. Therefore, studies around this CFH gene variant may be particularly informative for unique pathology involved in AMD. Inflammatory protein gene variants, such as interleukin 6 and the pro-inflammatory protein chymase, are associated with increased risk factor for AS [66, 87]. Although there is a lack of information specifically around CFH Y402H polymorphism and GN, the literature suggests an interesting connection between AMD, GN and CFH. After reviewing patient family history and performing genome linkage scans, it was found that the suggested MPGN risk locus (chromosome 1q31–32) physically resides near an AMD risk region (chromosome 1q-31) [5, 6]. This chromosome location is part of the region that harbours CFH genes and later has been suggested to be an AMD susceptibility locus [88]. Since there are at least eight common single nucleotide polymorphisms associated with AMD [88], it will be interesting to see if further CFH gene variations are shared between AMD and GN, which might direct future research around CFH genetics.

Other types of mutation in CFH and its related proteins suggest that CFH-related mutations might be worthwhile targets of research for both AMD and GN. A case study involving both AMD and MGPN showed the patient expressing an heterozygous amino acid substitution mutation of the CFH protein, namely the missense mutation in exon 9 of CFH (c.1292 G > A) [89]. In addition, the patient was homozygote for the CFH Y402H allele. After characterizing the subject’s CFH alleles at other positions, researchers concluded that this prototypical complement genetic profile might include a partial CFH deficiency and serve as major risk factors for both AMD and MPGN. Related to CFH function, a case study of poststreptococcal glomerulonephritis showed that a heterozygous single nucleotide insertion mutation in Complement Factor H–Related Protein 5 resulted in a premature stop codon and decreased protein levels in the serum [90]. The researchers speculate that this variant may be a risk factor for this type of GN.

The other major arm of complement, the classical complement pathway, seems to be involved primarily in AMD and AD, while some literature also support its involvement in AS and GN. The classical complement pathway appears to be involved in AMD pathogenesis by qPCR analysis that showed expression of classical pathway-related transcripts in choroid, RPE and neural retina [91]. The same study also suggested that cells in the choroid are the main sources of classical pathway gene expression [91]. Expression of C1 inhibitor factor in AMD supports this finding [92]. RNA studies show that while many classical pathway-related complement proteins are expressed at higher levels in AD brains, increase in C1q and C9 expression was particularly dramatic [93]. The sources of these complement proteins are found to be likely reactive glial cells and pyramidal neurons by in situ hybridization and immunohistochemistry experiments [94, 95]. In both AMD and AD, sources of classic complement components lie in the supporting cells. The current research of AMD focuses mainly on RPE cells. Shifting our attention to choroidal cells and classical complement activation maybe fruitful, especially in the area of wet AMD pathogenesis. Several protein markers in AD were reported to activate the classic complement pathway. Using direct in vitro evidence and indirect in situ evidence, Rogers and colleagues report that the cytotoxic Aβ binds and activates the classical complement pathway. They also report seeing these actions specifically localized to the brain areas where AD pathology occurs [96]. Later, molecular experiments showed that Aβ is capable of activating both the classical and alternative pathways [97]. Interestingly, the other important diagnostic protein of AD, tau, is also reported to activate the classical complement pathway [98]. Using both in vitro and in situ evidence, Shen and colleagues report that the pre-clinical presentation of both Aβ and tau protein may lead to chronic inflammation in AD [98]. Related to this finding in AD, Aβ is also found in the neuroretina [99]. Possibly, Aβ is capable of inducing macrophage recruitment and formation of membrane attack complex in the retina [99, 100]. Currently, our lab has conducted studies around the association of Aβ and classical pathway activation in AMD (unpublished data). In rodent models, anti-Aβ drug trials repeatedly relieved retinal deficits associated with AMD [101, 102].

Although there is a large body of literature supporting complement’s involvement in the pathogenesis of AMD, AD, AS and GN, the mechanisms by which the complement system induces damage are complex. Descriptions of the cascades leading to classic, alternative and leptin complement pathway activation in each disease are out of the scope for this review. For a review of complement system activation and possible relationship between AMD and AS, see [103]. A recent review by Heppner and colleagues nicely summarizes the inflammatory processes in AD [104]. Complement’s involvement in glomerular diseases is reviewed by [105].

### Macrophage recruitment

Besides direct cellular attack using membrane attack complex, one of the key functions of the complement pathway is to facilitate the phagocytosis of foreign pathogens by macrophages. Macrophages play a key role in AMD, AD, AS and GN and its recruitment rely on chemokines. In AMD, monocyte chemoattractant protein-1 (MCP-1), is found in high concentration in RPE cells [106]. MCP-1 acts in concert with other chemokines and cytokines to recruit macrophages and other immune cells to accumulate in areas surrounding drusen deposits. It is unclear if the arrival of macrophages is protective against AMD or facilitative of drusen formation. However, there is evidence supporting a correlation between the extent of macrophage phenotypic changes and the clinical stages of AMD [107, 108]. Since macrophages are closely associated with AMD disease progression, drawing understanding of macrophage recruitment and regulation from other related diseases might help to further understand their role in the development of AMD.

Microglia and peripheral macrophages are both associated with the progression of AD. Chronic activation of microglia is thought to induce neuron injury. Isolated proteins of neuritic and SP stimulate macrophages and microglia to secrete neurotoxins through a variety of cellular pathways, thus damaging neurons [80, 81]. Similar to the idea of stimulating complement activation with toxic proteins, stimulating macrophage recruitment in in vivo AMD models might yield understanding of macrophage chemoattractant profile in AMD. Interestingly, non-resident macrophages that infiltrate into the brain are suggested to have more phagocytic capacity than microglia, the resident macrophages in the brain [109]. Supporting the idea that peripheral macrophage must migrate into the disease site in order to exert its actions, the proinflammatory cytokine macrophage migration inhibitory factor (MIF) was found to be up-regulated in the cerebrospinal fluid of AD patients, suggesting that decreased capability of macrophage migration may contribute to AD [110]. Differentiation of microglia into macrophage-like cells relies on macrophage colony stimulating factor (M-CSF). Expression of this factor and its receptor is increased in a mouse model of AD and it appears to augment the effect of Aβ on microglia activation in in vitro experiments [111]. Unlike in AD, there is limited knowledge about the source of macrophages and its relation to AMD disease progression. Further study may be conducted to see whether choroidal macrophage, peripheral monocyte or retinal microglia have the most phagocytic capacity. Macrophage recruitment has been associated with glomerular injury in many diseases including GN. In immune mediated GN, macrophages become activated in the inflamed glomerulus and releases pro-inflammatory cytokines as well as ROS to cause glomerular basement membrane damage and fibrin deposition. Similar to AD, MIF was significantly up-regulated in a mouse model of autoimmune GN [112]. When these mice were genetically edited to have deficient MIF, renal macrophage recruitment and glomerular injury were significantly lowered [112]. Human studies also show evidence supporting the pathological role of MIF. In biopsies of GN patients, MIF expression was markedly increased in proliferative GN conditions [113]. This correlates to T-cell and macrophage infiltration in histological lesions. Combining with the evidence from AD studies, macrophage recruitment from the periphery is likely a compensatory mechanism to clear out toxic molecules. However, excessive macrophage activation and differentiation into M1/M2 subtype in AMD or foam cells in AS is clearly pathogenic.

Lipid accumulation inside macrophage is related to its differentiation. It has been suggested that defective ATP-binding cassette transporters in older macrophages result in accumulation of intracellular cholesterol, which then induce a phenotypic change from M1 pro-inflammatory macrophage to M2 pro-angiogenic macrophage [114]. After the change in macrophage phenotype, these cells produce different cytokines, express different receptors and perform different effector functions which may promote the transition from dry AMD to wet AMD [115]. In AS, lipoproteins cross into the arterial walls, bind proteoglycans and become oxidized to induce inflammation, macrophage recruitment and neovascularization. Arterial wall macrophages retain cholesterol-containing LDL through receptor mediated uptake or fluid-phase pinocytosis, which are reported to be very important events in atherosclerosis pathogenesis [23]. The two phenotypes of macrophages, M1 and M2, are found in atherosclerotic plaques and are distributed according to their functions [116]. Although there are many similarities between the lipid deposits in AMD and AS, it should be noted that the sources of the accumulated lipids differ. While the lipids in atherosclerosis come mostly from the plasma, the lipids in AMD may have a plasmatic or intraocular source [117, 118]. Given the contradictory roles of different types of macrophages, it is worthwhile to investigate the mechanism and regulation of macrophage recruitment and its further differentiation, in order to simultaneously harvest their benefits and prevent disease progression.

## Treatments for AMD, AD, AS and GN

Currently, there is ongoing research around the treatment for each disease discussed in this review. This review provides an overview of available treatments and potential new research directions pertaining to these diseases.

Current AMD treatments mainly focus on delaying disease progression. Although there are no effective treatments for early nonexudative AMD, nutritional supplements have been suggested to help protect against AMD progression. In the AREDS studies, supplementation with a combination of high dose vitamin C, vitamin E, beta-carotene, and zinc was shown to significantly decrease the odds for advanced AMD [119]. Follow up studies suggested that addition of lutein/zeaxanthin to the AREDS combination formula might be more appropriate than beta-carotene [120]. For the exudative form of AMD, approved treatments may include intravitreal anti-vascular endothelial growth factor injections or photodynamic therapy [121]. AD treatments mostly address symptoms without slowing the progression of disease or curative effects. Psychotropic medications treat the symptoms of AD by addressing specific secondary manifestations such as depression, agitation and hallucinations [122]. Newer AD treatment trials include monoclonal anti-Aβ antibodies, which did not show significant efficacy [123]. For a more in depth discussion about this treatment, please refer to [123]. Common treatments for AS include statins to lower LDL and cholesterol, fibrates to reduce triglycerides (TG), niacin to reduce TG and LDL. Treatment goals include lowering LDL cholesterol, non-HDL cholesterol, and ApoB, some of which cross over with the lipid molecules discussed in this review [124]. Recommended treatment for MPGN II involves corticosteroids to stabilize kidney function, plus both cyclophosphamide and mycophenolate mofetil to suppress the immune system [125]. Given this knowledge about the currently available treatments, there are other potential areas where investigation is likely worthwhile.

Since there are no current treatments for early non-exudative AMD and AD, it may be worthwhile to investigate the effect of adding ROS suppressing agents to the AREDS combination formula while simultaneously treating risk factors in these two disease models. ROS generation appears to be a common factor in various theories of AD pathogenesis and ROS suppression may be a promising method to better treat AD [126]. However, clinical trials of antioxidants have not yield fruitful results in treating AD [127]. Although antioxidants, such as the natural spice Curcumin, may not individually produce clinical efficacy, its combination with other therapies and its administration earlier in the disease history may warrant more research [128]. In AS, lipid lowering agents are established but not without their caveats. Although endogenous Coenzyme Q10 is the most active antioxidant in human, statin treatment have been found to decrease this natural defense mechanism [129, 130]. Investigations are warranted to solve this adverse interaction or more effort may be indicated to recommend dietary avoidance of oxidized cholesterols in commercially fried foods and polyunsaturated fatty acids. Drugs that resemble our endogenous antioxidant Coenzyme Q10 might be able to address the oxidized versions of cholesterol such as 7kCh or 24S–OHC discussed in this review. For apolipoproteins, drugs that lower ApoB levels are effective treatments for AS, but no treatment exists for lowering the subtypes of ApoE that are associated with AMD and AD. Since anti-Aβ antibodies demonstrated limited efficacy, one potential direction is to turn our research attention to pharmacological agents that target ApoE. By addressing Apo levels in the body, it may be possible to control the source of inappropriate inflammation and preserve appropriate inflammation for normal body responses.

## Conclusion

AMD, AD, AS and GN are related in many aspects. A general scheme of pathogenesis may be shared between the four diseases. Under macroscopic oxidative environments and microscopic ROS stress, lipids become oxidized and begin to deposit in the sites of disease development. These lesions cause an increased inflammatory response by activating the complement system as well as recruiting macrophages. As excessive inflammatory damage builds up, pathologic events occur in the eye, brain, blood vessels and kidneys. Shared genetic variations between the diseases further strengthen their connection and support common mechanisms of pathogenesis. By focusing on the similarities in lipid deposits and their consequences, this review reported common pathological mechanisms shared between AMD, AD, AS and GN, established a framework of ideas to aid understanding of AMD pathogenesis and provided novel strategies to search for more effective therapies.

## Abbreviations

24S–OHC: 24(S)-Hydroxycholesterol
25OH: 25-hydroxycholesterol
7kCh: 7-ketocholesterol
AD: Alzheimer’s disease
AGE: Advanced glycation endproduct
AMD: Age-related macular degeneration
ApoE: Apolipoprotein E
AS: Atherosclerosis
Aβ: Amyloid-β
BrM: Bruch’s membrane
CEP: Carboxyethylpyrroles
CETP: Cholesteryl ester transfer protein
CFH: Complement factor H
CSF: Cerebral spinal fluid
GBM: Glomerular basement membrane
GN: Glomerulonephritis
GO: Dicarbonyls glyoxal
LIPC: Hepatic lipase
MCP-1: Monocyte chemoattractant protein-1
M-CSF: Macrophage colony stimulating factor
MG: Methylglyoxal
MIF: Macrophage migration inhibitory factor
MPGN II: Membranoproliferative glomerulonephritis type II
MPGN: Membranoproliferative glomerulonephritis
oxLDL: Oxidized LDL
Ptau: Hyperphosphorylated tau protein
qPCR: Quantitative polymerase chain reaction
ROS: Reactive oxygen species
RPE: Retinal pigment epithelium
SP: Senile plaques
